# Neural computations in children’s third-party interventions are modulated by their parents’ moral values

**DOI:** 10.1038/s41539-021-00116-5

**Published:** 2021-12-17

**Authors:** Minkang Kim, Jean Decety, Ling Wu, Soohyun Baek, Derek Sankey

**Affiliations:** 1 Faculty of Arts and Social Sciences, The University of Sydney, Sydney, Australia; 2 Child Neurosuite, Department of Psychology, The University of Chicago, Chicago, USA; 3 Faculty of Information Technology, Monash University, Melbourne, Australia

**Keywords:** Morality, Education, Empathy

## Abstract

One means by which humans maintain social cooperation is through intervention in third-party transgressions, a behaviour observable from the early years of development. While it has been argued that pre-school age children’s intervention behaviour is driven by normative understandings, there is scepticism regarding this claim. There is also little consensus regarding the underlying mechanisms and motives that initially drive intervention behaviours in pre-school children. To elucidate the neural computations of moral norm violation associated with young children’s intervention into third-party transgression, forty-seven preschoolers (average age 53.92 months) participated in a study comprising of electroencephalographic (EEG) measurements, a live interaction experiment, and a parent survey about moral values. This study provides data indicating that early implicit evaluations, rather than late deliberative processes, are implicated in a child’s spontaneous intervention into third-party harm. Moreover, our findings suggest that parents’ values about justice influence their children’s early neural responses to third-party harm and their overt costly intervention behaviour.

## Introduction

Third-party intervention into perceived moral transgression is a key proximate mechanism enabling humans to manage conflict and maintain high levels of social cooperation^1,2^. *Intervention* (e.g. verbal reprimand, policing and protesting) is different from *punishment* or *defensive behaviours* in that it does not include retaliatory or punitive sanctions directed specifically at the wrongdoer^3,4^. However, intervention may often be costly, because the agent who intervenes can pay a personal cost while their actions yield no immediate personal gain^1^. Recent studies have shown that rudimentary abilities for costly intervention behaviours are present in infancy^5^ and toddlerhood^6,7^ well before children actually enact third-party intervention into real-life transgressions^8–10^. Although there is a growing body of literature on the development of children’s third-party intervention, little is known about the neural mechanisms underlying the early ontogeny of such behaviours. Furthermore, to our knowledge, there is no empirical work that has directly examined how child’s moral behaviours and their underlying neural processes are modulated by their parents’ values about justice. This study investigated the electrophysiological processing underlying children’s costly interventions and their time course, and how parents’ dispositions modulate the ontogeny of moral intervention behaviours.

Recent developmental work found that a preference for costly intervention behaviours emerges as early as 6 months in infancy^5^, and 20-month-old toddlers are willing to reward characters who intervene in third-party transgressions^6,7^. These findings suggest that the foundational capacity for third-party intervention, including normative understandings, emerge early in ontogeny. Beginning at around 3 years of age, young children do not just follow moral norms, but actively enforce them on others even when their own interests are not at stake^11^. For example, they show explicit disapproval and actively intervene against transgressions such as violations of property rights^8–10,12,13^, distribution inequity^14,15^ and physical harm^3^. Also, they often report (*tattle*) the perceived transgression to an authority on behalf of a third party^12,16,17^. Furthermore, intervention behaviours demonstrated by 3–6 years old children is nuanced, showing a higher probability of intervention into moral transgressions rather than violations of conventional norms and rules of games^16^. Many developmental psychologists believe that children’s protesting and reporting behaviours indicate that children have acquired a normative understanding against harm and inequity by around 3 years of age^18,19^.

Other scholars, however, cast doubt on whether preschoolers as young as 3 years of age can truly enact an altruistic reaction to transgression, arguing instead that what appears to be preschooler’s costly intervention behaviour is simply an indication of their impulsivity^20^, or their limited ability to consider the state of the transgressor’s emotions^21,22^, or an inability to anticipate future outcomes (e.g. retaliation, failing to complete a task) involving the self^23^. It has also been proposed that children’s third-party intervention behaviour is not primarily motivated by their intrinsic desire to enforce moral norms. Rather, the behaviours are more likely self-serving, for example, avoiding blame or maintaining reputation and trustworthiness within their affiliated group^14,24,25^.

Accumulating evidence undermines such scepticism. Children as young as 3 seem to intentionally enact third party intervention, even when they know it comes with personal cost. For example, they shut down a slide to prevent an antisocial child from playing on it, even when knowing they sacrifice their own opportunity to play on it^10^. Also, no significant correlation has been found between 3-year-olds’ third-party intervention and dispositional impulsivity^17^, suggesting that children’s intervention behaviours are not simply an impulsive ‘knee-jerk’ reaction. Moreover, many researchers point out that, when intervening in transgressions, children often use normative terms (e.g. “That’s wrong”, “You shouldn’t do that”), suggesting that young children may be “compelled to actually enforce” norms^11^ and promote good behaviours conducive to social cooperation^3,12,15^. However, whether and how children’s nascent understanding of social norms actually drives their costly intervention behaviours, cannot be understood by studying the behaviours of interest alone. Rather these need to be examined in conjunction with other independent measures^26,27^. Therefore, in this study, data from three independent sources were triangulated, namely: (a) electroencephalography (EEG), (b) young children’s behaviours recorded when involved in a transgression experiment and, (c) reports provided by the parents of the participating children of their child’s social and emotional dispositions.

The researchers conducting this study are aware it could be argued that when young children appear to be enforcing moral norms, they are simply imitating their parents, as children often do in other costly scenarios^28^. However, we question whether it is possible for children to feel compelled to imitate what they observed from parents, without ‘understanding’ what the parents were objecting to. In order to do ‘what parents did’ in a novel context, they would need to understand it was the violation of a somewhat universal *norm* that their parents were actually objecting to^11,29,30^. Previous studies have identified some strategies that parents adopt in moral induction. Parents treat moral violations (e.g. acts of harming) differently from children’s disobedience to social conventions^31^. For example, they often use firm-stern vocalization in response to moral transgression, and carefully explain why the act is wrong and direct the child’s attention to how specific acts might harm others’ rights or welfare^32^. However, it is not well understood how young children transfer moral norms learnt from parents to a novel contexts, where the parents are not present.

Meanwhile, some burgeoning work in neuroscience has begun to examine how parental dispositions are related to the neural processing of children’s moral judgement and behaviours. Some studies have shown that individuals’ disposition for justice sensitivity modulates their neural computations when they perceive interpersonal harm^33,34^. In the case of parents, their own moral values modulate the neural computations of their children when they perceive third-party social interactions^35^. In that latter study, parental justice sensitivity was predictive of infants’ and toddlers’ event-related potentials (ERP) differences in the perception of helping versus harmful scenes. ERP differences in a specific time-window (300 and 500 ms) were related to infants’ and toddlers’ reaching preference for a prosocial over an antisocial character shown after the EEG session. However, little is known about how parents’ dispositions, especially their concern for social injustice and empathy, shape children’s neural computations that subserve costly intervention into third-party moral transgressions. Therefore, this study specifically aimed to examine whether children’s implicit understanding of moral norms and values is modulated by parents’ justice sensitivity and empathy, such that it might predict their costly intervention behaviours.

Some handful of neuroscientific studies have started to build a neurobiological model of third-party intervention behaviours^36^. Regarding the *evaluation* of moral norm violation, EEG/ERPs studies show that an implicit assessment is made rapidly, within 200–250 ms after the onset of information that violates moral values and norms that one upholds^37–39^. For example, in a study with adults, a broadly distributed P2 was elicited by the onset of a target word, in a statement such as ‘euthanasia is acceptable’, when the target word ‘acceptable’ clashes with the reader’s value system^37^. Also, after 500–650 ms from the onset of a value-inconsistent word, another positive-going deflection (late positive potential, LPP) is observed^37,39^. This evidence shows that value-based judgement rapidly recruits attentional resources (as indexed by P2) and engages sustained processing within the affective system, as indexed by LPP^37,39^. Meanwhile, reduced P2 for the perception of harm/distress often indexes preclusion of sustained processing in prefrontal cortex, which may potentially lead to avoidant reactions^40^ or callous-unemotional reactions. Similar time-course of spatio-temporal responses have been detected in young children. Notably, children’s perceptual sensitivity and sustained attention to third-party moral transgressions is indexed by increased central P2 and LPP^41–43^.

There is a lack of consensus regarding how aversive reactions such as distress and anxiety, triggered by perceived norm violations, modulate subsequent prosocial actions. Some studies showed that personal distress stemming from perceived third-party harm does not motivate altruistic behaviours^44,45^, though, conversely, it can prohibit one’s costly interventions (i.e. self-concerned action or no action). One functional MRI study documented that personal distress, as indexed by the neural response in amygdala and periaqueductal grey does not predict costly altruism^46^. This network is known to trigger avoidant, withdrawal responses^47^. Another study found that empathic concern and costly donations were predicted by the neural response in the mesolimbic dopamine system, including the ventral striatum and ventromedial prefrontal cortex^48^. By contrast, other studies showed that aversive arousal guides behavioural choices in personal moral dilemmas. For instance, an EEG study with adult participants reported that individual dispositions in personal distress negatively predicted the mean percentages of utilitarian choices (e.g. killing one person to save more people) and positively correlated with the mean amplitudes of the P260, an ERP component reflecting an immediate emotional reaction during decision-making^49^. These results indicate that self-oriented feelings of anxiety and unease, rather than other-oriented feelings of concern, guide behavioural choices in personal moral dilemmas. Nevertheless, the jury is still out as to whether costly altruism is driven by an egoistic motivation to alleviate one’s own distress or by a genuine regard for another’s welfare^48^.

In ERP studies using stimuli evoking negative emotions, the frontal N2 (200–400 ms) is regarded as a marker of emotion regulation^50^. N2 is enhanced when participants engage with negatively valenced emotional evaluations of self and other^51^, and negative feedback concerning one’s performance^52^. The frontal N2 reflects the ‘augmentation of inhibitory controls’ when negative emotions arise^50^. In young children (4–6 yrs), N2 amplitudes are greater in the presence of negative emotion^53,54^, and more rapid when children have anxiety or fearful trait^53^. In a cross-sectional ERP study, Cheng and colleagues^55^ showed that the difference wave of N2 (third party pain vs. no pain), indexing inhibitory control over empathic arousal, has an age-related decrease between ages 3 and 9. If self-oriented feelings of anxiety and unease guide behavioural reactions^45^, heightened inhibitory control over personal distress, indexed by enhanced N2, may predict a child’s reaction to third-party moral transgressions.

**Fig. 1:**
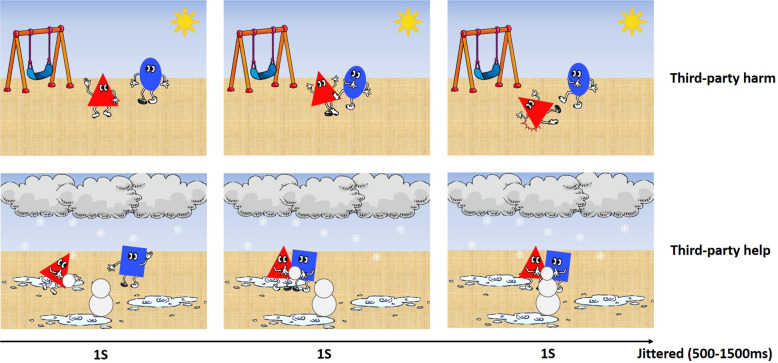
Two stimulus examples from the CMST. Top (Third-party harm): The blue character is intentionally kicking the red character, resulting in red character being knocked down. Bottom (Third-party help): The blue character is offering help to the red character who is moving a heavy snowball, resulting in the building of a snowman. The ERPs were time locked to the onset of the second image in the sequence.

Against the backdrop provided above, in order to elucidate how perceived norm violation and subsequent emotional arousal, elicited by observing third-party moral transgressions, lead to costly intervention in young children, we examined the relationship between children’s real-life costly behaviours and the spatio-temporal dynamics of neural processing recorded with EEG, in response to morally laden scenarios. *The* *Chicago* *Moral* *Sensitivity* *Task* (CMST, Fig. 1) comprises child-friendly visual scenarios depicting two characters intentionally performing either harming actions or helping actions^35,41^. As noted earlier, existing experimental paradigms have mostly relied on observing children’s overt behaviours or verbal responses, which are important yet limited in informing on how children’s implicit normative evaluation actually drives their costly intervention behaviours^26^. Meanwhile, neurodevelopmental studies, using EEG/ERP recordings, can contribute in elucidating the neural mechanisms that subserve attentional and affective processes initiated by the perception of norm violations, enabling us to understand how third-party costly intervention may be mediated by implicit moral evaluation during the early ontogeny of moral development^56,57^. Actually, a series of neurodevelopmental studies using the CMST^35,41,42^ indicated that there are distinctive contributions of both early automatic (e.g. central P2, frontal N2) and later controlled processes (e.g., central LPP) underlying children’s real-time evaluation of morally laden behaviours.

Based on the empirical work reviewed so far, we expected that children’s costly interventions in response to third-party harm would be initially driven by rapid implicit evaluation of norm violations. Specifically, we hypothesized that a difference between children who intervene in third-party harm and those who do not, might be identified by both early neural discrimination/recognition of moral norm violation (as indexed by central P2) and sustained evaluative processing (as indexed by frontal LPP). Additionally, we hypothesized that the decision to intervene in third-party harm will *not* be predicted by frontal N2 that reflects the inhibitory control over anxiety and distress. Testing a difference implicating the N2 response would help us elucidate whether children’s intervention behaviour is driven by impulsive reactivity^20^ or self-oriented feelings^49^. Finally, we hypothesized that parents’ dispositions in empathy and justice sensitivity would contribute to their children’s sensitivity to third-party moral transgressions, as indexed by ERPs data and their behavioural reactions in real-life settings.

## Results

### Children’s reactions to a third-party moral transgression

#### Protest behaviour

**Fig. 2:**
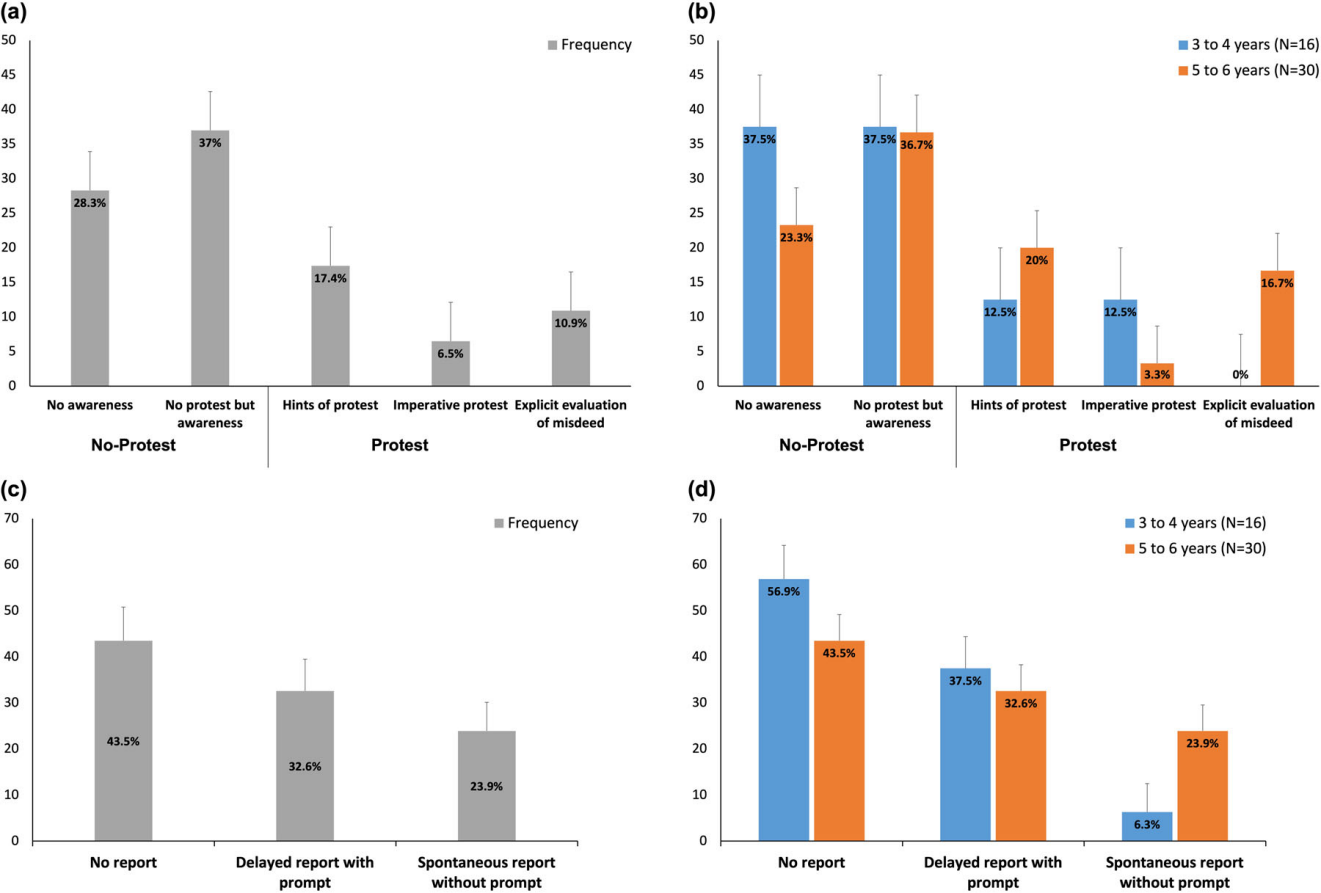
Percentages of protest and behaviours that fell into each category. The error bars show standard error for each type of protest and report.

Figure 2a shows percentages of protest responses that were observed. A total of 16 children (34.8%) explicitly protested to the adult wrong-doer while 30 children (65.3%) remained silent. Among protest behaviours, giving ‘hints of protest’ was the most prevalent form (*N* = 8, 50%), followed by ‘explicit normative evaluation of misdeed’ (*N* = 5, 31.3%) and ‘imperative protest (repeating the rule)’ (*N* = 3, 18.7%). Among those who did not protest, 17 children (56.7%) exhibited facial expressions of discomfort, uneasiness or anger, though these did not lead to protest behaviour. Coders could not identify any notable non-verbal signs from the remaining 13 children (43.3%), though they were all attentive to the adult transgressor’s behaviour. There was no correlation between the child’s age and the extent of protest shown (Spearman’s *ρ* = 0.082, *p* = 0.588). When children were divided into two age groups (3–4 years vs. 5–6 years, see Fig. 2b), there was still no statistical difference between two groups, *χ*^2^(4, *N* = 46) = 5.096, *p* = 0.278, though ‘explicit evaluation of misdeed’ was observable only in the older group. Child’s gender [*χ*^2^(1, *N* = 46) = 0.254, *p* = 0.61] and parent’s religious affiliation [*χ*^2^(1, *N* = 46) = 0.339, *p* = 0.560] did not associate with whether or not each child protested.

#### Reporting behaviour

Figure 2c shows percentages of report behaviours that were observed. A total of 26 children (56.6%) explicitly reported what they observed when the research assistant returned. There was a significant correlation between protest and report behaviours, *χ*^2^(8, *N* = 46) = 22.769, *p* = 0.004. Out of 16 children who protested to the transgressor, 14 children subsequently reported the misdeed. However, 2 children who had exhibited a hint of protest did not report what they had observed. Out of 30 children who did not protest, 18 children did not report what happened. However, 12 children reported what they witnessed, and 9 out of those 12 were those who exhibited non-verbal signs (e.g. discomfort, uneasiness or anger) when observing the transgressor’s misdeed. When children were divided into two age groups (3–4 years vs. 5–6 years, see Fig. 2d), there was no association between child’s age and the extent of report shown, *χ*^2^(2, *N* = 46) =4.301, *p* = 0.116. Child’s gender [*χ*^2^(1, *N* = 46) = 0.278, *p* = 0.598] and parent’s religious affiliation [*χ*^2^(1, *N* = 46) = 2.769, *p* = 0.096] did not correlate with whether or not each child reported.

### Relationship between children’s ERPs and moral behaviour observed in the experiment

In order to examine whether difference between children who intervene in third-party harm and those who do not, might be identified by different ERP patterns induced by moral norm violation, we conducted repeated-measures analyses of variance (ANOVA) in the pre-selected time-windows. In each analysis, the Grouping based on children’s reaction (Protester vs. Non-protester, Reporter vs. Non-reporter) was entered as a between-subject variable. The Condition within the CMST (Help vs. Harm) was entered as a within-subject variable.

#### Posterior N1 component (100–175 ms)

Between Protesters and Non-Protesters, there was no Group main effect on N1 amplitudes, *F*(1, 40) = 0.030, *p* = 0.862, power = 0.06, *f* = 0.032. Also, no Group × Condition interaction was found, *F*(1, 40)=0.126, *p* = 0.724, power = 0.06, *f* = 0.055. Between Reporters and Non-reporters, there was neither Group main effect on N1 amplitudes, *F*(1, 40) = 0.178, *p* = 0.675, power = 0.07, *f* = 0.063, nor Group × Condition interaction, *F*(1, 40) = 0.214, *p* = 0.646, power = 0.073, *f* = 0.071. These results indicate that there was no difference between Groups (Protester vs. Non-Protester; Reporter vs. Non-Reporter) during the initial attention and encoding process when observing visually presented stimuli.

#### Central P2 component (150–350 ms)

**Fig. 3:**
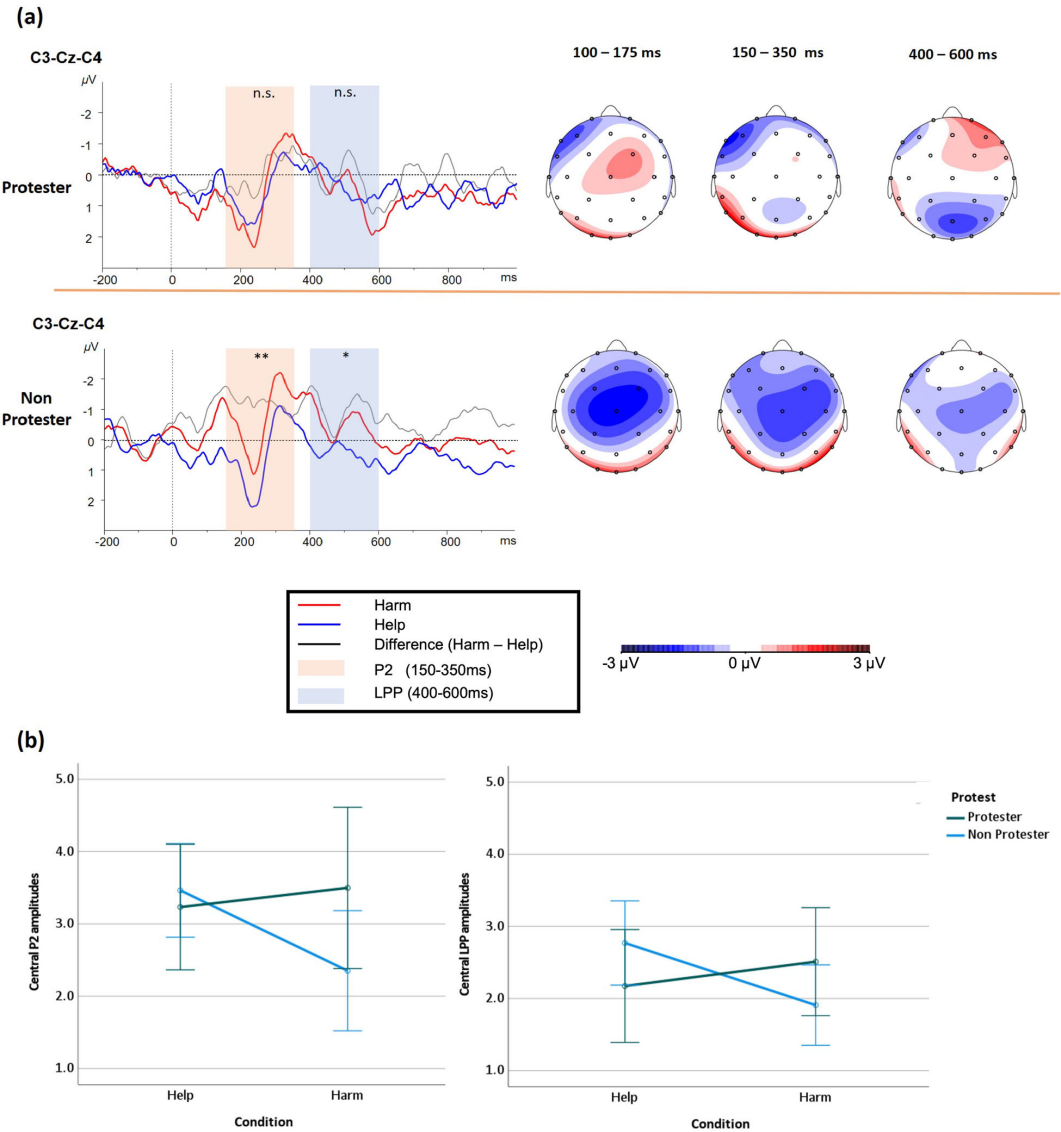
Comparisons of waveforms, difference waveforms and range-scaled voltage spline maps of the scalp distribution. **a** Left: Grand-averaged ERPs (P2 and LPP windows highlighted) in response to perceiving helping actions (blue) and harming actions (red) at the central cluster (C3-Cz-C4) across two groups (Protester vs. Non-Protester). Difference waveforms (grey) were generated by subtracting amplitudes of helping condition (blue) from harming condition (red), with negative values plotted upwards. Right: The range-scaled spline maps of the scalp distribution of three time windows in two groups. **b** Group (Protester vs. Non-Protester) × Condition (Help vs. Harm) interaction effects in Central P2 and LPP. The error bars show each group’s 95% CI in two different conditions.

In the entire sample, there was no Condition (Help vs. Harm) main effect, *F*(1, 40) = 2.291, *p* = 0.138, power = 0.327, *f* = 0.239. However, between Protesters (*N* = 15) and Non-Protesters (*N* = 27), there was significant Group × Condition interaction effect, *F*(1, 40) = 6.063; *p* = 0.018, power = 0.694, *f* = 0.39 (see Fig. 3). In the Non-Protest group, the mean P2 amplitude in response to viewing harming scenes (*M* = 2.358) was smaller than the mean amplitude of viewing helping scenes (*M* = 3.494), *t*(27) = 4.133, *p* < 0.001 < 0.05/2, with a medium effect size (power = 0.826, *d* = 0.568). In the Protest group, P2 amplitude in response to viewing harming scenes (*M*=3.568) was slightly bigger than helping scenes (*M* = 3.135), however, the difference was not statistically significant, *t*(14) = 0.481, *p* = 0.638, power = 0.08, *d* = 0.11. Between Reporters and Non-reporters, there was neither Group main effect on P2 amplitudes, *F*(1, 40) = 0.012, *p* = 0.915, power = 0.054*, f* = 0.028, nor Group × Condition interaction, *F*(1, 40) = 1.520, *p* = 0.225, power = 0.231*, f* = 0.193.

#### Frontal N2 Component (200–400 ms)

Between Protesters and Non-Protesters, there was no Group main effect on frontal N2 amplitudes, *F*(1, 40) = 1.545, *p* = 0.221, power = 0.236, *f* = 0.196. Also, no Group × Condition interaction was found, *F*(1, 40) = 0.009, *p* = 0.925, power = 0.05, *f* = 0.01. Between Reporters and Non-reporters, there was neither Group main effect on N2 amplitudes, *F*(1, 40) = 0.475, *p* = 0.495, power = 0.107, *f* = 0.11, nor Group × Condition interaction, *F*(1, 40) = 0.523, *p* = 0.474, power = 0.112, *f* = 0.115.

#### Central LPP Component (400–600 ms)

In the entire sample, there was no Condition (Help vs. Harm) main effect, *F*(1, 40) = 0.820; *p* = 0.371, power = 0.148, *f* = 0.142. However between Protesters and Non-Protesters, there was significant Group × Condition interaction effect, *F*(1, 40) = 4.286; *p* = 0.045, power = 0.545, *f* = 0.328 (see Fig. 3). In the Non-Protester group, the mean LPP amplitude in response to viewing harming scenes (*M* = 1.894) was smaller than the mean LPP amplitude of viewing helping scenes (*M* = 2.753), *t*(26) = −2.647, *p* = 0.014 < 0.05/2, power = 0.753, with medium effect size (Cohen’s *d* = 0.518). In the Protest group, the mean LPP amplitude from harming condition was slightly bigger (*M* = 2.530) than help condition (*M* = 2.163), though it was not statistically significant, *t*(13) = −0.670, *p* = 0.515, power = 0.095, *d* = 0.179. Between Reporters and Non-reporters, there was neither Group main effect, *F*(1, 40) = 0.597, *p* = 0.444, power = 0.122, *f* = 0.123, nor Group × Condition interaction, *F*(1, 40) = 0.600, *p* = 0.443, power = 0.122, *f* = 0.123.

### Relationship between children’s ERPs, costly moral behaviour and parents’ dispositions

**Fig. 4:**
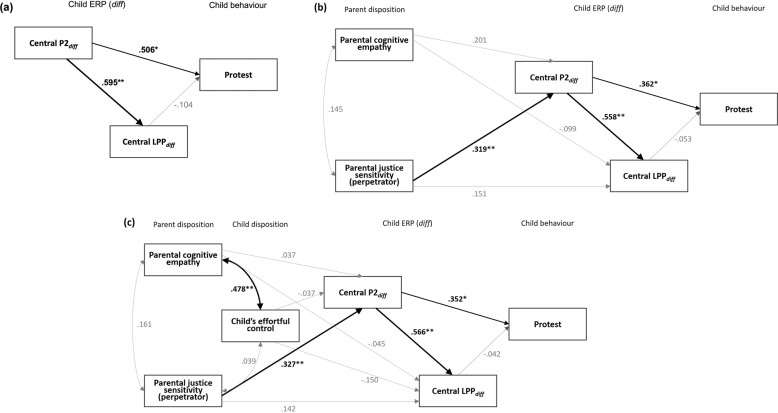
Standardized estimates for the effects included in the three models. **a** Model 1: Within-child (brain–behaviour) model (ppp = 0.44, power = 0.677). **b** Model 2: Intergenerational (Parent–child) model 1(ppp = 0.40, power = 0.803). **c** Model 3: Intergenerational (Parent–child) model 2 (ppp = 0.24, power = 0.795). Bold pathway delineates significant associations among primary variables of interest. ^*^Significant with a 90% Bayesian credible interval ^**^Significant with a 95% Bayesian credible interval.

In order to examine the relationships between parents’ dispositions (i.e. empathy and justice sensitivity), and their child’s costly moral behaviour and her/his EEG/ERP measures, Bayesian structural equation modelling (SEM) was conducted, utilizing AMOS 26^58^. Figure 4 illustrates the three models that were tested in this study.

The Model 1 (*within-child model*) represents brain–behaviour relationships that are implicated in child’s protest behaviour. With regard to selecting which ERP components to include in the Model 1, the hypothesized model included only central P2_diff_ and LPP_diff_, given that they were the only components that demonstrated a significant difference between two groups (Protester vs. Non-protester). The child’s age did not show any correlation with other variables (see Table 1), hence, the age was not included in this model and onwards. In addition to the variables from the Model 1, the Model 2 (*intergenerational model*) includes parent dispositions, namely caregiver’s empathy and justice sensitivity. Regarding caregiver’s empathy, based on previous work^41^ and observed zero-order correlations (Table 1), only cognitive empathy, not affective empathy, was included in the model. Among the four distinctive justice dimensions measured from the parents, ‘Perpetrator Sensitivity’ was included in the model, with its significant correlations with difference amplitudes for both P2 (*ρ* = 0.378, *p* < 0.05) and LPP (*ρ* = 0.290, *p* < 0.075) (see Table 1). Based on the literature indicating that a child’s dispositions moderate the relations between parenting and structure/function of neural networks that underpin children’s emotion regulation^59^, the Model 3 (*modified intergenerational model*) additionally included the child’s effortful control measured with the CBQ. Descriptive statistics and bivariate correlations for the primary study variables are provided in Table 1.

**Table 1:** Spearman’s rho (*ρ*) correlations and descriptive statistics for all variables.

		1	2	3	4	5	6	7	8	9	10	11	12	13	14	15
Child’s age	1. Age (months)	–														
Child’s dispositions	2. CBQ (Surgency)	−0.001	–													
	3. CBQ (Negative affect)	0.017	−0.129	–												
	4. CBQ (Effortful control)	0.159	−0.112	0.196	–											
Parent’s dispositions	5. Cognitive empathy	0.144	0.094	−0.041	0.439^**^	–										
	6. Affective empathy	−0.020	0.106	0.275	−0.069	0.449^**^	–									
	7. Victim sensitivity	−0.116	−0.029	0.218	0.022	−0.077	0.138	–								
	8. Observer sensitivity	−0.061	0.197	0.348^*^	0.011	0.277	0.610^**a^	0.293^*^	–							
	9. Beneficiary sensitivity	−0.141	0.067	0.372^*^	−0.154	−0.095	0.373^**a^	0.294^*^	0.603^**^	–						
	10. Perpetrator sensitivity	0.087	0.237	0.012	0.102	0.336^*^	0.334^*^	−0.206	0.258	0.201	–					
Child’s ERP amplitude difference	11. Frontal N2_diff_	0.110	−0.124	−0.125	−0.157	−0.079	0.094	0.041	0.004	0.006	−0.241	–				
	12. Central P2_diff_	0.095	0.147	0.248	0.116	0.157	0.280	−0.101	0.231	0.109	0.378^*^	0.058	–			
	13. Central LPP_diff_	0.013	0.051	0.411^**^	−0.118	−0.003	0.105	0.055	0.179	0.185	0.290^†^	0.053	0.576^**^	–		
Child’s protest behaviour	14. Child’s protest behaviour	0.082	−0.084	−0.046	0.110	0.201	−0.007	−0.055	0.036	−0.176	0.276^†^	0.129	0.350^*^	0.240	–	
Child’s reporting behaviour	15. Child’s reporting behaviour	0.193	−0.001	−0.102	0.399^**^	0.338^*^	−0.007	−0.172	0.070	−0.228	0.301*	−0.072	0.236	0.087	0.600^***^	–
	*M*	53.928	4.274	4.118	5.372	59.60	34.68	1.883	2.691	2.011	3.638	−0.890	−0.619	−0.433		
	SD	7.762	0.747	0.716	0.647	8.386	5.446	1.298	1.325	1.312	1.436	2.373	1.839	1.867		
	*N*	47	47	47	47	47	47	47	47	47	47	42	42	42	46	46

^†^*p* < 0.075; *p < 0.05; **p < 0.01; ***p < 0.001.

Note: Spearman’s rho (*ρ*) was calculated for the correlations between all variables including a categorical variable (protest and reporting behaviour) as *ρ* is less sensitive to outliners (Rousselet and Pernet, 2012).

^a^Parent’s affective empathy showed positive correlation with two other justice sensitivity scores (observer, beneficiary). However, parent’s affective empathy showed no correlation with children’s ERPs data and moral behaviours. Also, previous publications indicated that one’s cognitive empathy, not affective empathy, predicts children’s moral behaviour (Cowell and Decety, 2015). Therefore, in this study, only cognitive empathy, not affective empathy, was included in the structural equation modelling.

The posterior predictive value (ppp) for the Model 1 was 0.44, and the ppp values for Model 2 and 3 were 0.40 and 0.24, respectively, indicating the Model 1 (*within-child model*) has the best fit. Among the two intergenerational models, Model 2 demonstrated better fit, with smaller ppp value (ppp = 0.40, power = 0.803; see Table 2) compared to the Model 3 (ppp = 0.24, power = 0.795). Figure 4 presents all the paths included in the model test, with standardized regression weights. Significance levels are illustrated graphically by line thickness. In what follows, we discuss model pathways that directly address our primary research questions.

**Table 2:** Bayesian structural equation modelling results .

	Model 1 (ppp = 0.44)				Model 2 (ppp = 0.40)				Model 3 (ppp = 0.24)			
	Posterior mean	Standard deviation	90% Lower bound CI	90% Upper bound CI	Posterior mean	Standard deviation	90% Lower bound CI	90% Upper bound CI	Posterior mean	Standard deviation	90% Lower bound CI	90% Upper bound CI
*Focal path*												
Parental cognitive empathy → Central P2_diff_					0.042	0.034	−0.013	0.096	0.037	0.037	−0.023	0.097
Parental cognitive empathy → Central LPP_diff_					−0.022	0.030	−0.071	0.028	−0.010	0.033	−0.064	0.044
Parental justice sensitivity → Central P2_diff_					0.409	0.194	0.090	0.728	0.419	0.195	0.099	0.793
Parental justice sensitivity → Central LPP_diff_					0.196	0.180	−0.099	0.491	0.184	0.181	−0.114	0.480
Central P2_diff_ → Central LPP_diff_	0.604	0.138	0.377	0.831	0.567	0.149	0.323	0.811	0.575	0.147	0.334	0.816
Central P2_*diff*_ → Child’s protest	0.349	0.206	0.020	0.695	0.236	0.135	0.012	0.455	0.230	0.134	0.008	0.449
Central LPP_diff_ → Child’s protest	−0.071	0.197	−0.399	0.246	−0.034	0.135	−0.253	0.189	−0.027	0.134	−0.247	0.196
Child effortful control → Central P2_diff_									0.104	0.475	−0.680	0.881
Child effortful control → Central LPP_diff_									−0.433	0.419	−1.120	0.256
*Covariance*												
Parental cognitive empathy ↔ Parental justice sensitivity					1.838	2.237	−1.652	5.545	1.938	2.400	−1.786	5.996
Parental cognitive empathy ↔ Child effortful control									2.598	1.159	0.925	4.673
Parental justice sensitivity ↔ Child effortful control									0.036	0.183	−0.254	0.343

First, we sought to answer whether early neural discrimination of moral norm violation (as indexed by central P2) and sustained evaluative processing (as indexed by frontal LPP) predict young children’s intervention into a third-party moral transgression. After controlling for correlation between central P2_diff_ and LPP_diff_, only central P2_diff_ was directly associated with the child’s intervention into a third-party moral transgression. An increase in central P2 amplitude differences between harm and help conditions in the CMST was associated with child’s more explicit and evaluative response to adult’s moral transgression. Previously in this paper, it was reported that protesting children and their counterparts (non-protesters) showed a significant difference in central LPP via an ANOVA test. However, the output from the SEM shows that after controlling for LPP_diff_’s strong positive correlation with central P2_diff_, central LPP_diff_ (400–600 ms) does not predict the extent of a child’s behavioural response.

Second, we also examined associations between parental cognitive empathy and justice sensitivity dispositions and amplitudes difference from two ERP components. Parental justice sensitivity (perpetrator) showed a significant positive association with the child’s central P2_diff_. However, parental cognitive empathy showed no significant relationship with either P2_diff_ or LPP_diff_. In the Model 3, a child’s effortful control, as a stable disposition, showed a positive correlation with the parent’s cognitive empathy. However, child’s effortful control did not predict amplitude differences in either of the time windows. Moreover, as mentioned earlier, adding a pathway from a child’s effortful control to the Model 2 did not lead to better model fit.

## Discussion

Although existing literature documents that preschoolers intervene in third-party transgressions^8–10,12,13^, there is little consensus about what actually give rise to such actions. Does a child’s protest truly indicate her understanding of moral norms? Is the intervention behaviour driven by discomfort, or, perhaps the child simply imitates her parents’ actions? By triangulating data from three independent sources, including EEG/ERPs, children’s behaviours recorded when involved in a transgression experiment, and their parents’ observations, this study provides evidence that rapid implicit evaluations of norm violations are implicated in a young child’s intervention into third-party harm. Additionally, it indicates that parents’ values on justice sensitivity influence their pre-school aged children’s early neural responses to moral norm violations, which is then predictive of the child’s costly intervention behaviour.

Our study further provides evidence that children, as young as 3 years of age, can enact costly third-party intervention by protesting and reporting. Previous research has shown that young children from age 3 enact third-party punishment to transgressors shown in video or puppets^9,10^. In the present study, in the context of real-life transgression experiment, even the youngest participant (41 months old) engaged in costly intervention, by hinting disapproval to the adult transgressor (why are you doing that?) and subsequently reporting the damage when being prompted. During the experiment, confounding factors such as a sense of ‘responsibility’, were avoided by keeping the person playing the ‘research assistant’ role out of the room when the transgression occurred. Furthermore, when leaving the room, the ‘research assistant’ did not assign the children any special role to police or monitor the actions of the ‘visitor’ (who would transgress). Moreover, the transgressor was not an acquaintance of the child, and the book was said to belong to a university (not a child’s school nor researchers), hence giving little sense of in-group/out-group membership^11,60^. Also, the participating children would likely attribute ‘power’ and ‘authority’ to the visitor/transgressor, as an adult^26^. Nevertheless, in the real-life experimental context, 34.8% of children explicitly protested to the adult wrong-doer.

In line with our hypothesis, early neural discrimination of moral norm violation (as indexed by central P2) predicted children’s intervention in real-life moral transgressions. Importantly, non-protesting children exhibited significantly reduced P2 amplitudes for harming scenes. As noted earlier, in response to morally laden scenarios, centro-frontal P2 amplitude indexes automatic evaluative-categorization of information that violates moral values that one upholds^37–39^. In previous developmental studies using the CMST, P2 amplitudes were greater when perceiving harming actions than helping actions^42^. Furthermore, preschoolers who participated in an empathic learning intervention subsequently exhibited enhanced P2 in harming conditions^43^. Meanwhile, reduced P2 was observed when perceived negativity (or threat) quickly re-allocates available resources to sub-cortical regions in the preparation of ‘fight-flight responses’^47,61^, precluding sustained processing in prefrontal cortex^40^. Therefore, reduced P2 observed in non-protesting children implies that the extent of this preclusion, when viewing third-party transgression, is greater than in those who protested during the early time-course of neural processing. In protesting children, P2 amplitudes between harm and help scenes did not show a statistically significant difference. This may be due to the small sample size (*N* = 15). However, it might be also possible that the difference is more pronounced in older children. For example, in a previous study where P2 amplitude was larger when viewing harming actions than helping actions^42^, children’s average age was approximately 1 year older than the cohort of this study. Future work is warranted to elucidate the age-related development of P2 components when engaged with morally laden scenarios.

Contrary to our hypothesis that both P2 and LPP are implicated in third-party intervention behaviours, late regulatory processing, indexed by LPP, was not directly associated with a child’s costly actions. Although independent comparisons at each of the two time-window (i.e. P2 and LPP) indicate different patterns of neural computations between protesters and non-protesters, after controlling for a positive correlation between two ERP components, only P2 was directly associated with the child’s protest behaviour. This result implies that, at least in preschool age, the quality of initial implicit evaluation of moral norm violations has a strong sustained impact on later regulatory processes and, furthermore, it modulates subsequent decision-making to intervene. Additionally, the correlation across two time-windows and the identified predictive power of P2 suggests that claims for a pervasive dichotomic split between perception vs. action, and intuition vs. reasoning may misrepresent the processes that give rise to moral actions^62^^,^^63^. Rather, as noticed by other authors^37,63^, late neural dynamics are bounded by earlier temporal dynamics, meaning that salience detection and regulation processes are interwoven during the evaluation of moral transgressions and costly action.

This study adds to evidence that children’s third-party intervention is not driven by their impulsivity or an urge to release self-oriented feelings^49,64^. As shown above, there was no association between a third-party intervention (protest and report) and the augmentation of inhibitory control over negative emotion, indexed by N2. Also, similar to the previous findings^17^, children’s dispositional surgency (including impulsivity, tendency to approach and externalize) measured with the CBQ-VSF did not show any correlation with either behaviours (*ρ* = 0.129, *p* > 0.05 for protest, *ρ* = −0.072, *p* > 0.05 for report; see Table 1). Rather, the extent of children’s report behaviour showed a moderate correlation with their effortful control (*ρ* = 0.399, *p* = 0.005) and parent’s cognitive empathy (*ρ* = 0.338, *p* = 0.020). It might be possible that more sustained intervention behaviour, such as reporting, requires additional effortful control and perspective-taking, compared to the spontaneous protest to the transgressor. Therefore, the relationship between these variables needs to be further investigated and compared across different types of intervention behaviours that children engage with.

Finally, this study adds nuanced evidence on how parents shape children’s neurodevelopment that supports their moral decision-making. The study suggests that parents’ own values on justice modulate the early phase of neural computations of third-party moral scenarios in their children. However, this modulation was not observed with later controlled processes. This result adds to previous findings on parental modulation of moral development^32,41^ by providing new evidence that parents’ justice sensitivity is predictive of a child’s real-life moral behaviour, by shaping attentional and perceptual neural mechanisms. In other words, pre-schoolers of parents who are perceptive to harm or injustice inflicted by themselves, have not only developed a refined salience detection mechanism of third-party harm, but also are more likely to act against what they perceive to be another’s moral transgressions.

It should be emphasized that parent’s cognitive empathy was not implicated in the child’s neural computations of moral norms or their spontaneous intervention behaviour. However, parents’ cognitive empathy had a positive correlation with a child’s effortful control and their subsequent report behaviour. This distinct contribution made by two different dispositions (cognitive empathy and justice sensitivity) suggests that parenting strategies necessary to enhance a child’s moral development require both aspects: perspective-taking and understanding of moral values. Future studies that integrate functional neuroimaging methods and naturalistic observations of parent–child dyadic interactions need to be conducted in order to further elaborate the unique contributions from both parents’ empathy and justice values to children’s moral learning and development. Such studies would not only be of interest to developmental scientists, they would also have much to contribute to educational policy and practice, in providing a neurobiologically sound account of moral development^63^, leading to well-designed pedagogical strategies^43,65^.

We are aware that the relationships between spatiotemporal dynamics of neural processing and children’s costly intervention behaviours identified in this study are drawn from data at the group level. Also, due to the small sample size, analysis of ERP components, particularly the analysis of Protester group, were underpowered. Also, the participating children might have attributed ‘power’ and ‘authority’ to the adult transgressor^26^, preventing them from protesting and reporting even though they might have done so with a peer. Nevertheless, the findings from this data suggest potential avenues for future studies. For example, a follow-up study would be needed to examine the possible relationships between neural computations and costly behaviours using a *within-individual* approach to the brain–behaviour link^66^. Additionally, studies with different age groups and clinical populations, such as children with conduct problems or CU traits, would help elucidate whether the brain–behaviour link found in this study can be further generalized, and what factors might strengthen or weaken the pathways identified from this study.

## Methods

### Participants

A priori sample size was decided with a power analysis using G*Power 3.1.9.7. The necessary sample size for a large effect of *f* = 0.64, a 0.05 probability of error, and a power of 90% for a 2 (Group: Protester vs. Non-Protester) by 2 (Condition: Help vs. Harm) repeated-measure analysis of variance (ANOVA) was 28 (14 participants per group). The effect size of *f* = 0.64 was estimated from the observed effect size (*η*_p_^2^ = 0.29) reported in Meidenbauer et al. (2018)’s study^42^ on young children’s ERP responses to morally laden visual stimuli. In order to ensure a sufficient number of children with full data set (i.e. EEG, observed behaviours during the experiment, parent survey), we recruited 47 preschoolers between the ages of 41 and 72 months (*M* = 53.92 months, SD = 7.76; *N* = 16 girls) participated in the study. Participating children did not have any identified neurological (e.g. autism, ADHD) or other medical conditions. The average age of participating parents was 38.2 years old (SD=3.62; 41 mothers and 6 fathers). All responding parents were the child’s biological parent. At the time of the study, all responding parents reported they live with the child for 7 days per week. Except 2 parents, 45 parents reported they are the child’s primary caregiver. Although all participating pre-schools have multicultural intakes, all participating children had good English comprehension, with English as the compulsory language in the preschools they were attending. All children were born in Australia, and 26 children spoke English as home language (55.3%). 21 children used English as an additional language and their home language were Korean (*N* = 10, 21.3%), Mandarin (*N* = 9, 19.1%), Arabic (*N* = 1, 2.1%) and Thai (*N* = 1, 2.1%). Regarding the family’s religion, 24 parents (51.1%) responded they have no religious affiliation, followed by Christianity (*N* = 10, 21.3%), Catholic (*N* = 5, 10.6%), Islam (*N* = 4, 8.5%), Buddhism (*N* = 1. 2.1%), Coptic Orthodox (*N* = 1, 2.1%), Greek Orthodox (*N* = 1, 2.1%) and Hindi (*N* = 1, 2.1%).

Fliers introducing the study and inviting participation were distributed to directors of preschools, who forwarded them to prospective parents of children enroled in their preschool. Prior to giving consent, all parents were informed that at the end of the experiment their travel cost to the campus would be reimbursed and the child would be rewarded with a book ($20 equivalent) at the end of the experiment. Written informed consent was obtained from all parents and each child’s verbal assent was sought prior to commencing data collection. All data were collected in accordance with the ethical principles of the Declaration of Helsinki and the study was approved by the University of Sydney Human Research Ethics Committee (approval No. 2017/468).

### Third-party moral intervention task

This study adopted a design that was previously used to investigate children’s protesting and reporting third-party harm when an unfamiliar adult confederate intentionally damaged a library book^12^. Parents and children were informed that researchers would collect children’s drawings as a part of the project. After receiving parental consent and the child’s own verbal assent, a female research assistant invited a child to a drawing-room, equipped with a video camera. Children were given A3-size papers and coloured pens, then were told, “The first thing we ask all our visitors to do is draw a picture of a moment when you felt very grateful to somebody.” The research assistant encouraged and motivated the child, by drawing a picture of her own. The initial phase lasted until the child had settled in, relaxed and started her/his drawing.

Then, an adult female confederate knocked the door and entered, saying, “Hello. I came to participate in the study and was told that I should wait here, as the other rooms are locked.” The research assistant provided the confederate a seat beside the child and said, “Would *you* also like to draw a picture while waiting?” The research assistant then told both the child and the confederate, “I am going to leave to check the other room. I will leave my things (belongings) here: my books, pen and jacket. Oh, just one thing. Please be very careful with this book (attached with a university logo and barcode) because it belongs to the library and*I*have to take good care of it. I need to return it tomorrow.” The research assistant ensured this remark was heard and understood by the child, checking her/his facial expression.

After the research assistant left the room, the transgression session started. The confederate picked up the book, saying: “Oh, this looks like an interesting book. I think I will read this book instead of doing the drawing.” After skimming through a few pages, the confederate said, “Oh, I really like the picture on this page. I’m going to tear this page out of the book, the librarian won’t know if only one page is missing.” Then, she removed the page from the book in full sight of the child and put the torn page into her pocket, so that it would not be noticed by the research assistant.

During the last phase of the transgression, the research assistant returned, telling the confederate, “Hey, the other room is now open. You can go to the other room.” The confederate immediately left the room, pretending nothing had happened. During the subsequent minute, the research assistant simply resumed her drawing without saying anything. This period offered the child an opportunity to spontaneously report the transgression she/he had observed to the research assistant. If the child had not spontaneously reported the transgression, the research assistant asked the child, “Was everything OK? Please tell me what happened while I was away.”

In accordance with the approved ethics protocol, a debriefing was followed once data collection was complete. At the debriefing, the main aim of the session was clarified to both parents and children. Each child was told that the person who they met was acting out a scenario, and emphasized that books borrowed from the library should be taken care of, so taking pages out of a book is not allowed. After the debriefing, parents were given an opportunity to decide whether they would like to have the child’s data included in the study. Nobody wished to withdraw their child’s data from the study.

The video recordings were analysed with a coding scheme adapted from the previous studies^9,12,26^. The analysis focused on: (i) whether the child immediately *protested* to the adult confederate, and (ii) whether the child later *reported* what he/she saw to the researcher. Details of codes and rating methods are provided below alongside the data analysis procedure.

### CMST and EEG recording

The CMST comprised three-scene cartoon scenarios depicting characters intentionally helping (e.g., helping another character making a snowman, helping another character after a fall) or intentionally harming another (e.g., kicking another character, destroying another character’s toy blocks) (see Fig. 1). These scenarios were validated with an independent group of American children between the ages of 3 and 8 years^41^. The version of the CMST used in this study included 120 trials (60 helping and 60 harming), 3 s in duration per scenario, randomly presented via E-Prime 2 (professional), with a jittered inter-trial interval of 500–1500 ms^35,41^. In both conditions (Helping vs. Harming), the shapes and colours of characters were counterbalanced. ERPs were time-locked to the onset of the second picture. EEG data were collected from a 32-channel active electrode system (actiCHamp+Brain Vision Recorder, Brain Products, Germany) at 2 kHz, referenced to Cz, and all electrodes impedances were kept below 30 kOhms.

### Questionnaires

Children’s social and emotional disposition were collected with the *Very Short Form of the Children’s Behaviour Questionnaire* (CBQ-VSF)^67^. The CBQ-VSF is a 36-item observer-report questionnaire, representing three broad dimensions of temperament observable in young children: (a) Surgency (12 items, *α* = 0.58), (b) Negative Affect (12 items, *α* = 0.53) and (c) Effortful Control (12 items, *α* = 0.62). Parents rated the degree to which each item, reflecting a type of behaviour, applied to his/her child over the past six months on a 7-point Likert scale (1 = extremely untrue to 7 = extremely true; NA = does not apply).

Parents’ dispositional empathy was assessed with the *Questionnaire of Cognitive and Affective Empathy* (QCAE)^68^. The QCAE is a 31-item self-report questionnaire, measuring (a) Cognitive Empathy (19 items, *α* = 0.90) and (b) Affective Empathy (12 items, *α* = 0.77). Items were rated by level of agreement using a 4-point Likert scale (1 = strongly disagree to 4 = strongly agree).

Parents’ values on justice were assessed with the *Justice Sensitivity Short Scales* (JSSS)^69^. The JSSS measures justice sensitivity from the Victim (2 items, *α* = 0.79), Observer (2 items, *α* = 0.70), Beneficiary (2 items, *α* = 0.78) and Perpetrator (2 items, *α* = 0.85) perspectives. Response options for the justice sensitivity scales raged from 0 (*totally disagree*) to 5 (*totally agree*).

### Data analysis

#### Coding and analysis of children’s reaction

Children’s protest reactions were coded into five different categories and scores were assigned as illustrated in Table 3. Reliability on a random 25% of the sample was excellent (*κ* = 0.95 for whether or not a child protested; *κ* = 0.78 for the sub-category assigned for a child).

**Table 3:** Coding scheme for protest behaviours.

Category	Codes	Behavioural indicators
Protest	Explicit normative protest	Child intervenes with explicit evaluation, using normative vocabulary (‘No, it’s not OK to rip out [remove a page from] someone else’s book’, ‘it’s not right’)
	Imperative protest (Repeating the rule)	Child commands to stop action, in reference to the rule (‘No, you don’t do that’ or ‘you may not do that’; ‘You can’t. You’ll get in trouble’; ‘She said she should take care of the book’; ‘No! Don’t tear it!’)
	Hints of protest	Child protests but clear attribution to the other two categories is not possible; includes using a protesting tone of voice in exclamations (‘Hey!), questions (‘Why are you doing that?’), or statements (‘That’s A’s book’; ‘That’s from the library’; ‘I’ll tell A’)
No Protest	No protest but awareness	Child’s face or non-verbal behaviours show his/her concerns or discomfort (e.g. frowning, stopping the drawing, staring at the confederate)
	No awareness	Child shows neither any awareness nor any sign of protest

Children’s reports were coded into three categories as described in Table 4. Reliability on a random 25% of the sample was excellent (*κ* = 1.00).

**Table 4:** Coding scheme for reporting behaviours.

Category	Coding	Behavioural indicators
Report	Spontaneous report without prompt	As soon as the research assistant returns, the child tells her that the visitor took out a page from the book. The voice tone conveys concerning or complaining or disapproving tone.
	Delayed report with prompt	The child tells the research assistant that the visitor took out a page only after being prompted by her question (e.g. Was everything OK when I was out?)
No report	No report	The child does not report what he/she observed until the end of the session.

#### ERPs data analysis

EEG data pre-processing was performed with Brain Vision Analyser 2.0 software (Brain Products). Data were down-sampled offline (256 Hz) and referenced to the average of all electrodes. Following referencing, EEG data were filtered using an IIR filter of 1–30Hz. Artefact rejection was carried out using a −150 to 150 μV threshold and visual inspection. Gratton-Coles ocular artefact correction was performed on EEG data to identify and correct ocular movements, seeded off electrode Fp1^70^. Following basic EEG pre-processing, data were segmented according to trial type (harming/helping). Epochs were created with 200 ms baselines and 1000 ms stimulus presentation following the onset of the second stimulus frame. Epochs were averaged by trial type and were baseline corrected. Corrected averages per individual were combined for a grand average per individual and ERPs were analysed across subjects by trial types and reactions shown at the experiment (protest vs. non-protest). Only children who had at least 20 artefact-free trials for each trial type (Harming and Helping) were included in the grand average and further analyses (*N* = 42).

In line with the hypotheses, the following latency windows were investigated: the posterior N1 (100–175 ms), the central P2 (150–350 ms), the frontal N2 (200–400 ms) and the central LPP (400–600 ms). Posterior N1, though not directly related to hypotheses, was tested in order to rule out group differences stemming from initial attention and early encoding of visual information^71,72^. For each region, three electrodes were included in the averaging and analyses: the frontal region included F3, Fz, F4; the central region included C3, Cz, C4; the posterior region included P3, Pz, P4 sensors. In order to avoid inflating Type, I error rate, the choice of region and electrodes was made a priori, based on existing literature^41–43,50,71,72^. Within each region, chosen three electrodes to have relatively less influence from potential artefacts (e.g. eye and facial muscle movements) and least overlap from adjacent regions. Given no statistical difference in amplitudes across three sites within each region, amplitudes were averaged for each region. With our interest in understanding how the implicit evaluation of norm violations might contribute to protesting and reporting activities, we focused our analyses on the difference of mean amplitude values in two conditions (Help vs. Harm), then compared the extent of difference between the two groups (Protester vs. Non-Protester; Reporters vs. Non-Reporters) using a repeated measure analysis of variance (ANOVA). Greenhouse–Geisser corrections were applied when assumptions of sphericity were not met. For ANOVAs, Cohen’s *f* was used to measure effect sizes. Post-hoc comparisons or simple effect analyses were performed with a Bonferroni’s correction and Cohen’s *d* was reported.

#### Bayesian structural equation modelling

In order to provide a comprehensive account of how parents’ dispositions are linked to individual differences in the neural computations implicated in costly moral behaviour, structural equation modelling (SEM) was conducted, utilizing AMOS 26^58^. In this analysis, only Protest behaviour was included as Report behaviour did not show any association with child’s ERP data. For ERP data, we focused our analyses on the difference waves generated by subtracting the helping from the harming condition (i.e. P2_diff_ = P2_Harm_–P2_Help_). Given that the child’s protest behaviour is categorical data that does not follow the normal distribution, Bayesian SEM was undertaken in order to test three models: (a) Within-child (brain–behaviour) model that includes child’s ERP and protest behaviour only (Model 1); (b) intergenerational model that adds parent’s dispositions to the child’s brain–behaviour model (Model 2); c) refined intergenerational model that adds child’s disposition to the second model (Model 3). Model parameters were estimated using the AMOS 26 Bayesian method that uses Markov-chain Monte Carlo (MCMC) sampling to generate full posterior distributions of model parameters. We have set up 500 burn-in and tried 25,000 MCMC runs after burn-in. i.e., 25,500 total MCMC run. Regarding prior, we adapted diffuse priors for all parameters with the default prior distribution in AMOS 26, i.e., uniform from –3.4 × 10^−38^ to 3.4 × 10^38^ to each parameter^58^. Regarding the model fit test, posterior predictive *p*-values (ppp) was considered^73^. There is some agreement that ppp values close to 0.50 imply a good fit while values <0.05 may indicate poor fit^73,74^.

### Reporting summary

Further information on research design is available in the Nature Research Reporting Summary linked to this article.

## Supplementary information

Reporting Summary

## Data Availability

The datasets presented in this article are not readily available because ethics to place data in a public repository was not obtained from the children and families who participated in this study (HREC Approval No. 2017/468). The data may be made available on request to the authors after review of the request by the Human Research Ethics Committee (HREC). Requesters should submit a data analysis plan before requesting the data. Requests to access the datasets should be directed to the corresponding author.

## Code availability

In this study, no custom scripts were used for the analysis. Syntax and commands used in licensed software are available at https://osf.io/p5b8u/?view_only=b9e677c0fa8e42e0ac15d51f24d0fa4e.
