# Supplementary material for: Neural computations in children’s third-party interventions are modulated by their parents’ moral values
Source: NPJ Sci Learn. 2021 Dec 17;6:38. doi: 10.1038/s41539-021-00116-5 (PMC8683432; doi:10.1038/s41539-021-00116-5)
Supplement: Supplementary file 1 — Reporting Summary [file 41539_2021_116_MOESM1_ESM.pdf]

nature portfolio

Corresponding author(s): Minkang Kim

Last updated by author(s): Sep 28, 2021

Reporting Summary

Nature Portfolio wishes to improve the reproducibility of the work that we publish. This form provides structure for consistency and transparency in reporting. For further information on Nature Portfolio policies, see our Editorial Policies and the Editorial Policy Checklist.

Statistics

For all statistical analyses, confirm that the following items are present in the figure legend, table legend, main text, or Methods section.

n/a	Confirmed	
☐	☒	The exact sample size (n) for each experimental group/condition, given as a discrete number and unit of measurement
☐	☒	A statement on whether measurements were taken from distinct samples or whether the same sample was measured repeatedly
☐	☒	The statistical test(s) used AND whether they are one- or two-sided<br>*Only common tests should be described solely by name; describe more complex techniques in the Methods section.*
☒	☐	A description of all covariates tested
☐	☒	A description of any assumptions or corrections, such as tests of normality and adjustment for multiple comparisons
☐	☒	A full description of the statistical parameters including central tendency (e.g. means) or other basic estimates (e.g. regression coefficient) AND variation (e.g. standard deviation) or associated estimates of uncertainty (e.g. confidence intervals)
☐	☒	For null hypothesis testing, the test statistic (e.g. F, t, r) with confidence intervals, effect sizes, degrees of freedom and P value noted<br>*Give P values as exact values whenever suitable.*
☐	☒	For Bayesian analysis, information on the choice of priors and Markov chain Monte Carlo settings
☒	☐	For hierarchical and complex designs, identification of the appropriate level for tests and full reporting of outcomes
☐	☒	Estimates of effect sizes (e.g. Cohen's d, Pearson's r), indicating how they were calculated

Our web collection on statistics for biologists contains articles on many of the points above.

Software and code

Policy information about availability of computer code

Data collection	EEG data recording: Brain Vision Recorder (Brain Products)<br>CMST presentation: E-Prime Professional (Psychology Software)<br>Video recording of experiments: Panasonic HC-V110
Data analysis	EEG preprocessing: Brain Vision Analyzer 2.0 (Brain Products)<br>ANOVAs and correlation analysis: IBM SPSS Statistics Ver 27.0<br>SEM analysis: IBM AMOS Ver 26.0<br>Effect size and power analysis: G*Power 3.1.9.7

For manuscripts utilizing custom algorithms or software that are central to the research but not yet described in published literature, software must be made available to editors and reviewers. We strongly encourage code deposition in a community repository (e.g. GitHub). See the Nature Portfolio guidelines for submitting code & software for further information.

Data

Policy information about availability of data

All manuscripts must include a data availability statement. This statement should provide the following information, where applicable:

- Accession codes, unique identifiers, or web links for publicly available datasets
- A description of any restrictions on data availability
- For clinical datasets or third party data, please ensure that the statement adheres to our policy

The datasets presented in this article are not readily available because ethics to place data in a public repository was not obtained from the children and families who participated in this study (HREC Approval No. 2017/468). The data may be made available on request to the authors after review of the request by the Human

March 2021

Research Ethics Committee (HREC). Requesters should submit a data analysis plan before requesting the data. Requests to access the datasets should be directed to the corresponding author.

Field-specific reporting

Please select the one below that is the best fit for your research. If you are not sure, read the appropriate sections before making your selection.

☐ Life sciences ☒ Behavioural & social sciences ☐ Ecological, evolutionary & environmental sciences

For a reference copy of the document with all sections, see nature.com/documents/nr-reporting-summary-flat.pdf

Behavioural & social sciences study design

All studies must disclose on these points even when the disclosure is negative.

Study description	This study comprises quantitative (continuous+ordinal) measures from electroencephalography (EEG) recording, a live interaction experiment (quasi experimental design), and a parent survey.
Research sample	Forty-seven preschoolers from Sydney, Australia (M = 53.92 months, SD = 7.76; N=16 girls) participated in the study. Participating children did not have any identified neurological (e.g. autism, ADHD) or other medical conditions. The average age of participating parents was 38.2 years old (SD=3.62; 41 mothers and 6 fathers). All responding parents were the child's biological parent. Further details are provided in the paper under 'Participants' section.
Sampling strategy	A priori sample size was decided with a power analysis using G*Power 3.1.9.7. The necessary sample size for a large effect of f= 0.64, a 0.05 probability of error, and a power of 90% for a 2 (Group: Protester vs. Non-Protester) by 2 (Condition: Help vs. Harm) repeated-measure analysis of variance (ANOVA) was 28 (14 participants per group). The effect size of f=.64 was estimated from observed effect size (ηp2 = 0.29) reported in Meidenbauer et al. (2018)'s study on young children's ERP responses to morally-laden visual stimuli. For the recruitment, fliers introducing the study and inviting participation were distributed to directors of preschools, who forwarded them to prospective parents of children enrolled in their preschool. There was no additional sampling within those volunteered to participate in the study.
Data collection	1. Instruments included in the Parent Survey: Very Short Form of the Children's Behaviour Questionnaire (CBQ-VSF), Questionnaire of Cognitive and Affective Empathy (QCAE), Justice Sensitivity Short Scales (JSSS)<br>2. EEG task: The Chicago Moral Sensitivity Task (CMST)<br>3. Procedures of Third-party moral intervention task: Fine details of this procedure are provided in the paper under the heading of 'Third-party moral intervention task'.
Timing	Data was collected between November 2017 and June 2018 without any gap period.
Data exclusions	Out of total 47 participants, only children who had at least 20 artifact-free trials for each trial type (harming and helping) were included in the EEG grand average and further analyses (N=42). With the given pre-determined criterion, 5 participants were excluded from the analysis where EEG data is included.
Non-participation	No participants withdrew their consent. All who consented actually participated in the study.
Randomization	No randomization was required in the given design.

Reporting for specific materials, systems and methods

We require information from authors about some types of materials, experimental systems and methods used in many studies. Here, indicate whether each material, system or method listed is relevant to your study. If you are not sure if a list item applies to your research, read the appropriate section before selecting a response.

Materials & experimental systems

n/a	Involved in the study	
☒	☐	Antibodies
☒	☐	Eukaryotic cell lines
☒	☐	Palaeontology and archaeology
☒	☐	Animals and other organisms
☐	☒	Human research participants
☒	☐	Clinical data
☒	☐	Dual use research of concern

Methods

n/a	Involved in the study	
☒	☐	ChIP-seq
☒	☐	Flow cytometry
☒	☐	MRI-based neuroimaging

Human research participants

Policy information about studies involving human research participants

Population characteristics	See above.
Recruitment	Fliers introducing the study and inviting participation were distributed to directors of preschools located in various suburbs in Sydney. These suburbs were largely classified as mid-range socio-economic status areas (3-4 out of maximum 5) according to the Australian Relative Socio-economic Advantage and Disadvantage (IRSAD) SEIFA scores. Directors of those preschools forwarded fliers to prospective parents of children enrolled in their preschool. Prior to giving consent, all parents were informed that at the end of the experiment their travel cost to the campus would be reimbursed and the child would be rewarded with a book ($20 equivalent) at the end of the experiment. Written informed consent was obtained from all parents and each child's verbal assent was sought prior to commencing data collection.
Ethics oversight	The University of Sydney Human Research Ethics Committee

Note that full information on the approval of the study protocol must also be provided in the manuscript.
